# *Levilactobacillus brevis* with High Production of Putrescine Isolated from Blue Cheese and Its Application

**DOI:** 10.3390/ijms24119668

**Published:** 2023-06-02

**Authors:** Yuta Ami, Narumi Kodama, Masahiro Umeda, Hanae Nakamura, Hideto Shirasawa, Takashi Koyanagi, Shin Kurihara

**Affiliations:** 1 Faculty of Biology-Oriented Science and Technology, Kindai University, Kinokawa 649-6493, Wakayama, Japan; 2 Faculty of Bioresources and Environmental Sciences, Ishikawa Prefectural University, Nonoichi 921-8836, Ishikawa, Japan

**Keywords:** polyamine, putrescine, food, blue cheese, *Levilactobacillus brevis*

## Abstract

Polyamine intake has been reported to help extend the lifespan of animals. Fermented foods contain high concentrations of polyamines, produced by fermenting bacteria. Therefore, the bacteria, isolated from fermented foods that produce large amounts of polyamines, are potentially used as a source of polyamines for humans. In this study, the strain *Levilactobacillus brevis* FB215, which has the ability to accumulate approximately 200 µM of putrescine in the culture supernatant, was isolated from fermented foods, specifically the Blue Stilton cheese. Furthermore, *L. brevis* FB215 synthesized putrescine from agmatine and ornithine, which are known polyamine precursors. When cultured in the extract of Sakekasu, a byproduct obtained during the brewing of Japanese rice wine containing high levels of both agmatine and ornithine, *L. brevis* FB215 grew to OD_600_ = 1.7 after 83 h of cultivation and accumulated high concentrations (~1 mM) of putrescine in the culture supernatant. The fermentation product also did not contain histamine or tyramine. The Sakekasu-derived ingredient fermented by the food-derived lactic acid bacteria developed in this study could contribute to increasing polyamine intake in humans.

## 1. Introduction

Polyamines are hydrocarbon compounds with two or more amino groups in their molecular structure and are present in cells of almost all living organisms, from prokaryotes to higher plants and animals [1]. Polyamines contribute to the stabilization of genomic DNA [1], inhibit aberrant methylation [2], are involved in gene transcription and translation [3,4], and promote cell differentiation [5]. There are three sources of polyamines in the human body, namely, oral polyamine intake [6], polyamine biosynthesis by intestinal microbiota [7,8], and polyamine biosynthesis by a host cell. The ability to biosynthesize polyamines decreases with age in animals, such as rats [9,10,11], but controlling polyamine biosynthesis is currently difficult. On the other hand, the oral intake of polyamine-rich foods increases blood polyamine levels in humans [12], and putrescine produced by intestinal bacteria in the large intestinal lumen are imported into the colonic tissue [13]. Therefore, regulating the intake of polyamines derived from food and intestinal microbiota would be effective in controlling the polyamines level in the body. In previous animal studies, wherein the lifespan extension effect was confirmed, among others, the polyamine concentration in high-polyamine diets was four [14] and ten times [15,16] higher than that in low-polyamine diets. In contrast, the dose in a recent randomized controlled human trial of polyamine intake with malt-derived supplements was 1.2 mg/day [17], which is only 4%, 7.5%, 4.6%, and 3.3% of the 29 mg [18], 16 mg [19], 26 mg [20], and 36 mg [21] daily polyamine intakes in the USA, Turkey, Japan, and Sweden, respectively. This is because only a few food ingredients contain high concentrations of polyamines [6,22]. Therefore, it is essential to develop new foods that contain high concentrations of polyamines, which are safe and easy to consume. Some fermented foods contain high concentrations of polyamines [6] that could be derived from fermenting microorganisms. If the bacteria producing high concentrations of polyamines are isolated from food-derived fermenting microorganisms, supplements containing high concentrations of polyamines could be obtained from culture supernatants. In addition, if new fermented foods are prepared using the isolated bacteria, polyamine intake could be increased. Furthermore, if this novel fermented food is ingested orally without sterilization, the polyamine-producing bacteria in the fermented food will reach the large intestine, and polyamines production in the large intestinal lumen can be expected, as previously reported [23].

Blue cheese is characterized by the presence of N-methyl ketones produced from fatty acids, with a strong flavor resulting from diverse lipolysis [24]. During blue cheese production, the raw milk is coagulated with rennet extract and then fermented, mainly with *Penicillium roqueforti* [24]. Pasteurized blue cheese contains 1.1 µg of agmatine, 18 µg of putrescine, and 10 µg of spermidine/g of wet weight [25].

*Levilactobacillus brevis*, isolated in this study as a polyamine-high-producing bacterium, is a type of lactic acid bacteria associated with the production of fermented foods, such as kefir [26], pickles [27], and sauerkraut [28]. *L. brevis* is known to produce *γ*-aminobutyric acid, which exhibits antihypertensive and antidepressant effects and is used as a probiotic [29]. Regarding polyamine metabolism and transport in *L. brevis*, *L. brevis* encodes an active agmatine deiminase and amino acid transporter that efficiently exchange agmatine and putrescine [30].

Sakekasu, a byproduct of the Japanese rice wine brewing process, has long been consumed in Japan. Moto, a starter of Japanese rice wine brewing, contains 0.41 mg/g ornithine [31], and Japanese rice wine contains 0.11 mg/g agmatine [22], which are precursors of polyamines. Therefore, Sakekasu could contain high levels of agmatine and ornithine. If these putrescine precursors could be converted to putrescine, new foods with increased polyamines concentration could be developed.

In this study, *L. brevis*, a major producer of putrescine isolated from the blue cheese, was cultured in Sakekasu extract to produce food ingredients with high concentrations of putrescine.

## 2. Results

### 2.1. Screening of High-Putrescine-Producing Bacteria from Blue Cheese

**Figure 1:**
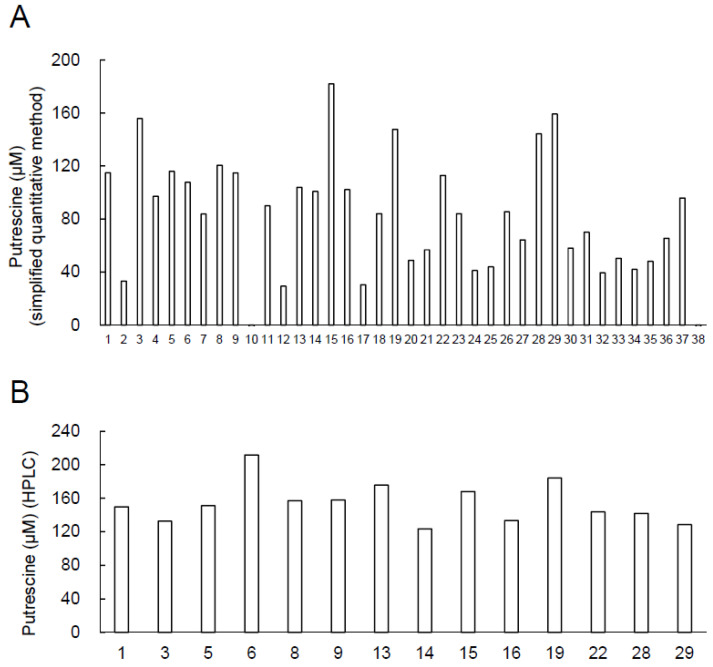
Putrescine concentration of culture supernatants of bacteria isolated from blue cheese. (**A**) The putrescine concentration of the culture supernatants of the isolated 38 strains was measured using a simplified quantitative method (PuO-POD-4AA-TOPS method described previously [32]); (**B**) The culture supernatant of the 14 strains that contained more than 100 µM putrescine in the culture supernatant was quantified by HPLC.

The growth of 38 bacterial strains isolated from blue cheese in this study in MRS broth was between OD_600_ ≈ 1 and 5 (Supplementary Figure S1). The concentration of putrescine in the culture supernatants was determined (Figure 1A) using a simplified quantitative method (PuO-POD-4AA-TOPS method described previously [32]). Simplified quantification of putrescine indicated that the culture supernatant of strains 1, 3, 5, 6, 8, 9, 13, 14, 15, 16, 19, 22, 28, and 29 contained >100 µM of putrescine. Polyamine quantification in the culture supernatants of these 14 strains was performed using HPLC.

### 2.2. Putrescine Concentration of Culture Supernatants of Candidate of the High-Putrescine-Producing Bacterial Strains

HPLC analysis demonstrated that the culture supernatants of 14 candidates of the high-putrescine-producing bacterial strains contain 124–184 µM of putrescine (Figure 1B).

### 2.3. Estimation of Bacterial Species by 16S rDNA Sequencing

Sequencing of 16S rDNA was performed on a 500 bp partial sequence containing the V1-V2 region in the 14 bacterial strains. The BLAST search of the nucleotide sequence revealed that the partial sequence of 16S rDNA of 12 strains, namely, 1, 5, 6, 8, 13, 14, 15, 16, 19, 22, 28, and 29, showed 100% identity with that of *L. brevis*, and the partial sequence of 16S rDNA of strains 3 and 9 was 100% identical to that of *Lactiplantibacillus plantarum*. Strain No. 13 was designated as *L. brevis* FB215. The almost entire (1466 bp) 16S rDNA sequence of *L. brevis* FB215 (accession number LC768903) was found to be 100% identical to the 16S rDNA sequence of *L. brevis*. Microscopic examination of *L. brevis* FB215 after Gram staining revealed the bacterium to be rod-shaped and partially chained (Supplementary Figure S2).

### 2.4. Effect of the Addition of Putrescine Precursors to the Medium on the Putrescine Productivity of L. brevis FB215

**Figure 2:**
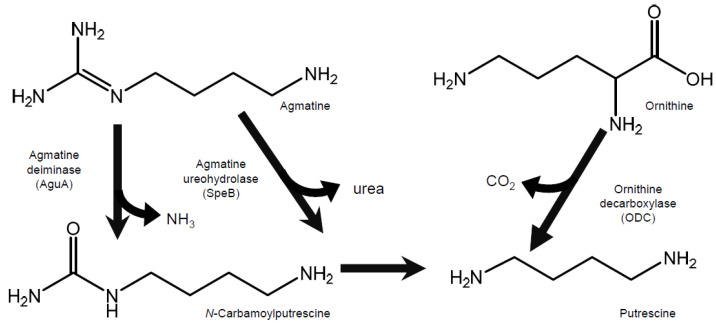
The previously reported putrescine biosynthetic pathways (3157043).

**Figure 3:**
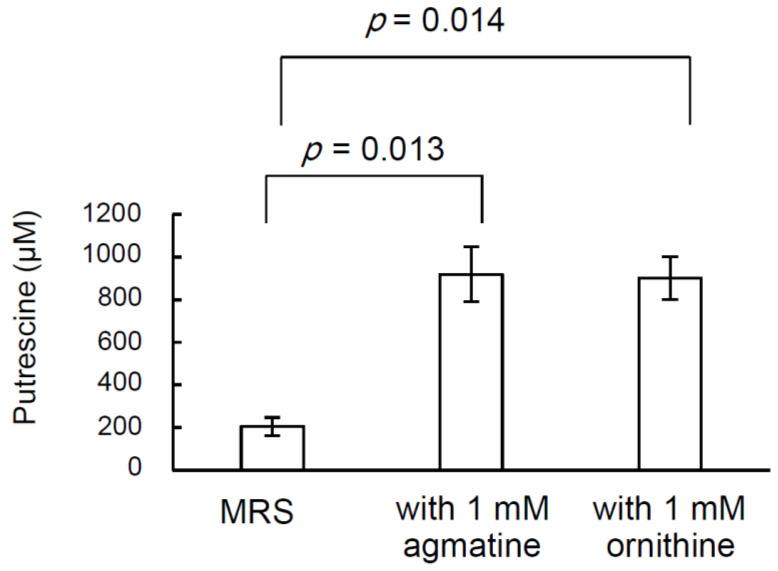
Production of putrescine when *Levilactobacillus brevis* FB215 is grown in MRS broth supplemented with putrescine precursors. *L. brevis* FB215 was inoculated in 50 mL of MRS broth supplemented with ornithine or agmatine at a final concentration of 1 mM and incubated under anaerobic conditions for 48 h at 37 °C. At the end of the cultivation period, the polyamine concentration in the culture supernatant was determined using HPLC (*n* = 3). Two-tailed *t*-tests were performed.

Two pathways for bacterial putrescine synthesis have been reported, whereby one produces putrescine from agmatine and the other from ornithine [33] (Figure 2). To analyze the effect of the addition of putrescine precursors to a medium on the production of putrescine by *L. brevis* FB215, the concentration of putrescine in the culture supernatant of *L. brevis* FB215, grown in MRS broth supplemented with ornithine or agmatine at a final concentration of 1 mM, was quantified using HPLC. After 48 h of cultivation, the putrescine concentration in the culture supernatant of the one with MRS broth without putrescine precursors was 205 µM, whereas the concentrations of putrescine in the culture supernatant grown in MRS broth supplemented with 1 mM ornithine and 1 mM agmatine were 918 µM and 901 µM, respectively (Figure 3).

### 2.5. Determination of Ornithine and Agmatine Concentrations in Sakekasu Extract

Concentrations of agmatine, ornithine, and putrescine in the Sakekasu extract were determined as approximately 2 mM, 1.3 mM, and 90 µM, respectively (Supplementary Figure S3).

### 2.6. Cultivation of L. brevis FB215 in Sakekasu Extract

**Figure 4:**
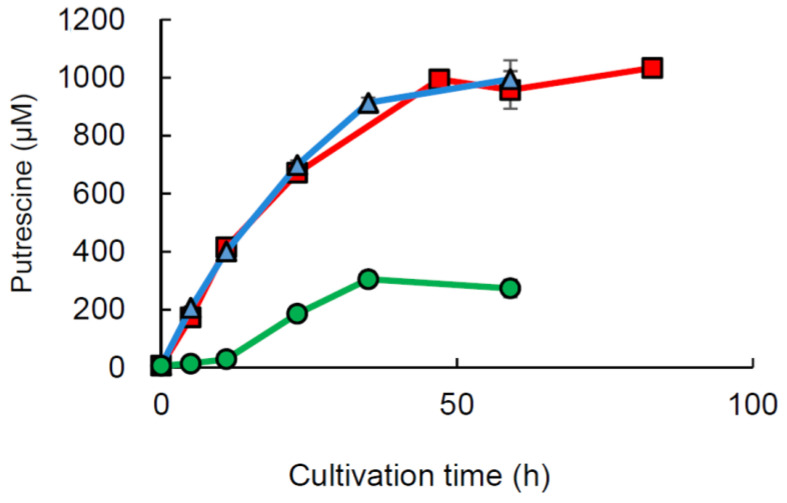
Putrescine concentration of the culture supernatant of *Levilactobacillus brevis* FB215 grown in Sakekasu extract. *L. brevis* FB215, *S. epidermidis* FB146 [34], and *L. curvatus* KP3-4 [23] were inoculated in Sakekasu extract and incubated at 37 °C. Polyamine concentrations in culture supernatants at different growth phases were determined. The levels of putrescine in the culture supernatants of *L. brevis* FB215, *S. epidermidis* FB146, and *L. curvatus* KP3-4 are represented by red squares, blue triangles, and green circles, respectively (*n* = 3).

*L. brevis* FB215 was cultured in Sakekasu extract. The OD_600_ value was measured over time, and the polyamines concentration in the culture supernatant was determined. For comparison, *Latilactobacillus curvatus* KP3-4 [23] and *Staphylococcus epidermidis* FB146 [34], which were previously reported as high-putrescine-producing bacteria, were cultured in Sakekasu extract. The results showed that *L. curvatus* KP3-4, *S. epidermidis* FB146, and *L. brevis* FB215 reached a maximum OD_600_ of approximately 0.2 at 59 h, 0.6 at 59 h, and 1.7 at 83 h after inoculation (Supplementary Figure S3), respectively, and the culture supernatants contained 304, 994, and 1033 µM of putrescine, respectively (Figure 4). The pH of the culture supernatants of *L. curvatus* KP3-4, *S. epidermidis* FB146, and *L. brevis* FB215 in the growth and stationary phases was measured and found to decrease with bacterial growth (Supplementary Figure S4B). Although cadaverine was detected (Supplementary Figure S5D), neither histamine, agmatine, spermidine, and spermine (Supplementary Figure S5D) nor tyramine (Supplementary Figure S5G) were detected in the supernatant of *L. brevis* FB215 cultured in Sakekasu extract for 95 h.

## 3. Discussion

In this study, *L. brevis* FB215, a lactic acid bacterium that produces high levels of putrescine, was isolated from blue Stilton cheese. A simplified quantitative method indicated that 14 of the 38 strains isolated from the blue Stilton cheese in this study produced more than 100 µM of putrescine in the culture supernatant (Figure 1). When the V1–V2 regions of 16S rDNA were analyzed, they were all 100% identical to the 16S rDNA of *L. brevis* or *L. plantarum*. This suggests that *L. brevis*, or the closely related *L. plantarum*, which is a major producer of putrescine, is present at high levels in the blue Stilton cheese. *L. brevis* FB215 produced 4–5 times higher levels of putrescine in the culture supernatant with either agmatine or ornithine, the precursors of putrescine, added to the medium than without (Figure 3). Therefore, *L. brevis* FB215 likely possesses both a pathway for the conversion of agmatine to putrescine, catalyzed by agmatine ureohydrolase (SpeB) and/or agmatine deiminase (AguA), and a pathway for the conversion of ornithine to putrescine, catalyzed by ornithine decarboxylase (SpeC and/or SpeF) (Figure 2). The reported high-putrescine-producing fermenting bacteria *L. curvatus* KP 3-4 [23] and *S. epidermidis* FB146 [34], encoded only the ornithine decarboxylase gene, and supplementation of the media with agmatine did not affect putrescine production in these strains. Since *L. brevis* FB215 converts both ornithine and agmatine precursors to putrescine, this bacterium could be useful for fermenting ingredients containing ornithine and putrescine simultaneously to produce a food product with high concentrations of putrescine.

In Sakekasu extract containing both ornithine and agmatine, *L. brevis* FB215, isolated from the blue cheese, grew up to eight times more than our previously reported putrescine-producing lactic acid bacteria *L. curvatus* KP 3-4 [23] (Supplementary Figure S4A). *L. brevis* FB215 accumulated approximately 3 times the concentration of putrescine (~1 mM) in the supernatant compared to *L. curvatus* KP 3-4 [23] (Figure 4). The low putrescine productivity of *L. curvatus* KP 3-4 may be due to the low growth level of this strain (Supplementary Figure S4A). Although the supernatant of *L. brevis* FB215 did not contain spermidine (Supplementary Figure S5), previous studies have shown that putrescine, produced by intestinal bacteria in the colonic lumen, is converted to spermidine in the intestinal tissue [13] and increases blood spermidine levels [35]. Therefore, the ingested putrescine can be converted into spermidine in vivo, providing various health benefits associated with spermidine.

Tyramine and histamine are biogenic amines that are harmful to human health and are often produced during the ripening of cheese; thus, the concentrations in food products are restricted [36,37]. As *L. brevis* FB215 isolated in this study was a bacterium isolated from a ripened cheese, Blue Stilton cheese, there was concern about the productivity of these amines. However, the fermentation product of Sakekasu extract by *L. brevis* FB215 did not contain tyramine and histamine. This eliminated one of the potential hazards of the fermentation product of Sakekasu extract by *L. brevis* FB215.

Polyamine loadings in previous studies, reporting the health-promoting effects of polyamine ingestion in mice, are summarized below. In references [14,15,16], the low polyamine group was fed on 20, 10, and 21 μg/g body weight/day (μg/g/day) polyamines, and the high polyamine group was fed on 70, 100, and 210 μg/g/day polyamines (Table 1; body weight, food intake, and water consumption were approximated from the previous study [38]). In a human study, the polyamine consumption is estimated at 0.48 and 0.50 μg/g/day in the group without and with polyamine intervention [17], respectively, assuming a subject weight of 60 kg and a normal polyamine intake [18] of 29 mg/day. This shows that the baseline levels of polyamine intake and loading in mouse experiments and human epidemiological studies were significantly different.

In contrast, a previous study showed that consuming 45–90 g of Natto, a fermented soybean food, per day (polyamine equivalent of 16–32 mg) increased the average level of spermine in the blood by 1.1 times [12]. In this previous study [12], the polyamine intake of 0.48 μg/g/day in the normal diet was increased to 0.75 μg/g/day by Natto loading. Therefore, consuming tens of milligrams of polyamines per day could have health benefits, such as anti-inflammatory effects. If 500 mL of Sakekasu extract fermented by *L. brevis* FB215 and derived from blue cheese is consumed, 45.5 mg of putrescine will be loaded. Since the daily intakes of polyamines in Europe, Turkey, America, and Japan are reported at 42 [39], 16 [19], 29 [18], and 26 mg/day [20], respectively, the consumption of fermented Sakekasu extract by *L. brevis* FB215 can increase polyamine intake by 108%, 284%, 157%, and 175%, respectively.

## 4. Materials and Methods

### 4.1. Isolation of Polyamine-High-Producing Bacteria from the Blue Cheese

Commercially available Blue Stilton cheese, chopped with kitchen scissors sterilized by spraying with 70% ethanol, was mixed with sterile phosphate-buffered saline (PBS) to produce a 10% (*w/v*) suspension. The suspension was diluted 10^−2^, 10^−3^, 10^−4^, and 10^−5^ times with sterile PBS. The serially diluted suspension (100 µL) was spread on antibiotic-free MRS plates and incubated with an AnaeroPack (Mitsubishi Gas Chemical, Tokyo, Japan) at 37 °C for 120 h under anaerobic conditions. Single colonies of 38 strains on the MRS plate were inoculated into 500 µL of MRS broth, then anaerobically incubated with the AnaeroPack at 37 °C for 48 h under anaerobic conditions. The culture was stored as frozen bacterial glycerol stocks at −80 °C.

### 4.2. High-Throughput Determination of Putrescine Concentration in Culture Supernatants of the Blue Cheese-Derived Bacteria

Frozen bacterial glycerol stocks of the blue cheese-derived bacterial strains were inoculated into 500 µL of MRS broth aliquoted into sterile 96-well deep-well plates (Thermo Scientific Nunc, Tokyo, Japan), and incubated at 37 °C under anaerobic conditions using the AnaeroPack for 48 h. The culture medium was then centrifuged at 1900× *g* for 20 min, and the putrescine concentration of the culture supernatant was determined in a simplified manner using the PuO-POD-4AA-TOPS method [32].

### 4.3. Determination of Polyamines by HPLC

A total of 14 bacterial strains, whose putrescine concentration in the culture supernatant exceeded 100 µM in the previous section, were inoculated into 500 µL of MRS broth and incubated anaerobically with the AnaeroPack at 37 °C for 24 h. The culture medium was centrifuged (2700× *g*, 15 min), and the 200 µL of culture supernatant was mixed with 20 µL of 100% TCA to precipitate the proteins in the culture supernatant. The precipitated proteins were removed by centrifugation (18,700× g, 5 min), the supernatant was filtered through a Cosmonice Filter W (Merck, Darmstadt, Germany), and the polyamine concentration was analyzed using a the previously described HPLC system (Chromaster, Hitachi Ltd., Tokyo, Japan) equipped with a cation exchange column (#2619PH, 4.6 × 50 mm; Hitachi) [34].

### 4.4. Analysis of 16S rDNA

The bacterial cells were disrupted by vigorous agitation with glass beads and centrifuged to obtain a supernatant containing bacterial chromosomal DNA. Using the chromosomal DNA as a template, PCR was then performed with primers 1510R (5′-ACGGYTACCTTGTTACGACTT-3′) and 7F (5′-AGAGTTTGATYMTGGCTCAG-3′) by a KOD FX Neo DNA polymerase (TOYOBO, Osaka, Japan) to amplify 16S rDNA. The PCR products were purified using the Wizard^®^ SV Gel and PCR Clean-Up System (Promega, Madison, WI, USA) and subjected to the 16S rDNA sequencing. The determined sequences were confirmed using BLAST, and the bacterial species were estimated.

### 4.5. Gram Staining

Gram staining was performed with a standard protocol, using Victoria blue for first staining, followed by destaining with ethanol containing picric acid and counterstaining with safranin.

### 4.6. Culture in MRS Medium Supplemented with Putrescine Precursor

The frozen bacterial glycerol stock was streaked onto an MRS plate and anaerobically incubated at 37 °C for 48 h using the AnaeroPack. The resulting single colony was inoculated into 50 mL MRS broth and incubated anaerobically at 37 °C for 24 h, using the AnaeroPack to obtain pre-culture. Approximately 2 µL of the pre-culture was inoculated using a copy plate 96 (Tokken, Kanagawa, Japan) of MRS broth or MRS broth supplemented with L-Ornithine HCl (Peptide Institute, Inc., Osaka, Japan) or Agmatine Sulfate (Tokyo kasei Kogyo Co., Ltd., Kanagawa, Japan) at a final concentration of 1 mM. The culture was incubated anaerobically at 37 °C for 48 h using the AnaeroPack. The culture was centrifuged (1900× *g*, 20 min), and the putrescine concentration of the supernatant was determined by HPLC.

### 4.7. Agmatine and Ornithine Concentration in Sakekasu Extract

In total, 10 g of Sakekasu (Kitaoka syuzo, Yoshino, Japan) stored at −20 °C was mixed with 40 mL of deionized water and suspended. The suspension was centrifuged at 18,700× *g* for 5 min, and the supernatant was sterilized by autoclaving to obtain Sakekasu extract.

Protein in the 200 µL of Sakekasu extract was precipitated by adding 20 µL of 100% TCA and centrifuged at 18,700× *g* for 5 min. The resulting supernatant was filtered through a Cosmonice Filter W. The agmatine concentration in Sakekasu extract was determined using an HPLC system (Chromaster, Hitachi Ltd., Tokyo, Japan) equipped with a cation exchange column (#2619PH, 4.6 × 50 mm; Hitachi, Tokyo, Japan) [34]. The ornithine concentration in Sakekasu was analyzed using an amino acid analyzer (No. L-8900, Hitachi Ltd., Tokyo, Japan) equipped with an ion-exchange column (No. #2622SC, 4.6 × 60 mm, Hitachi Ltd., Tokyo, Japan) [31]. The eluted ornithine was detected and quantified using the post-column ninhydrin labeling method.

### 4.8. Cultivation of Bacteria in Sakekasu Extract

Frozen bacterial glycerol stock was streaked onto MRS plates and anaerobically cultured at 37 °C for 68 h. The resulting single colonies were inoculated into 3 mL of MRS broth and statically cultured at 37 °C for 24 h to obtain a preculture. The preculture was then inoculated into 8 mL or 14 mL of Sakekasu extract adjusted to pH 7 to give an initial OD600 of 0.03. Cultures were incubated statically at 37 °C, and growth (OD_600_) was measured at 5, 11, 23, 35, 47, 59, 71, 83, and 95 h after inoculation. The culture supernatant was centrifuged (18,700× *g*, 5 min), and the concentration of putrescine in the supernatant was determined by HPLC.

### 4.9. Determination of Production of Amines by L. brevis FB215 in Sakekasu Extract

The presence of putrescine, cadaverine, spermidine, spermine, and histamine was determined by HPLC equipped with the #2619PH column, as described above [34]. The presence of tyramine was determined by a reversed-phase HPLC equipped with a Discovery HS F5 column (4.6 × 250 mm) (Sigma-Aldrich/Supelco, Bellefonte, PA, USA), as previously described [40].

### 4.10. Measurement of pH

The pH of the culture of Sakekasu extract was measured using a pH meter (Twin pH Meter II LQUAtwin, Asone, Osaka, Japan).

## 5. Conclusions

A highly polyamine-producing lactic acid bacterium, *L. brevis* FB215, was isolated from blue cheese. Unlike our previously reported bacteria, this strain produced putrescine from both agmatine and ornithine. The fermentation product of Sakekasu extract with *L. brevis* FB215 contained 1 mM of putrescine and did not contain histamine or tyramine. Consumption of Sakekasu extract fermented by food-derived lactic-acid bacteria *L. brevis* FB215 is expected to contribute to improved health through increased polyamine intake in humans.

## Figures and Tables

**Table 1:** Polyamine loadings in previous studies.

Animals	Low Polyamine Group (μg/g Body Weight/Day)	High Polyamine Group (μg/g Body Weight/Day)	Administration Method	Literature
Mouse	19.66 ^a^	73.58 ^a^	diet	[14]
Mouse	10.44 ^b^	104.40 ^b^	*ad libitum* drinking water	[15]
Mouse	11.23 ^b^	112.34 ^b^	*ad libitum* drinking water	[16]
Human	0.48 ^c^	0.50 ^c^	supplement	[17]
Human	0.48 ^d^	0.93 ^d^	fermented soy bean (Natto)	[12]

^a^ Values were calculated using previously reported food intake and body weight of mice [38]. ^b^ Values were calculated using previously reported water intake and body weight of mice [38]. ^c^ Human body weight was assumed to be 60 kg. ^d^ Polyamine levels in Natto were calculated from previous reports [6], and human body weight was assumed to be 60 kg.

## Data Availability

All data needed to evaluate the conclusions in this work are present in the paper.

## Supplementary Materials

The following supporting information can be downloaded at: https://www.mdpi.com/article/10.3390/ijms24119668/s1.
