# Supplementary material for: Levilactobacillus brevis with High Production of Putrescine Isolated from Blue Cheese and Its Application
Source: Int J Mol Sci. 2023 Jun 2;24(11):9668. doi: 10.3390/ijms24119668 (PMC10253434; doi:10.3390/ijms24119668)
Supplement: Supplementary file 1 [file ijms-24-09668-s001.zip › ijms-2385349-supplementary.pdf]

Supplementary Materials

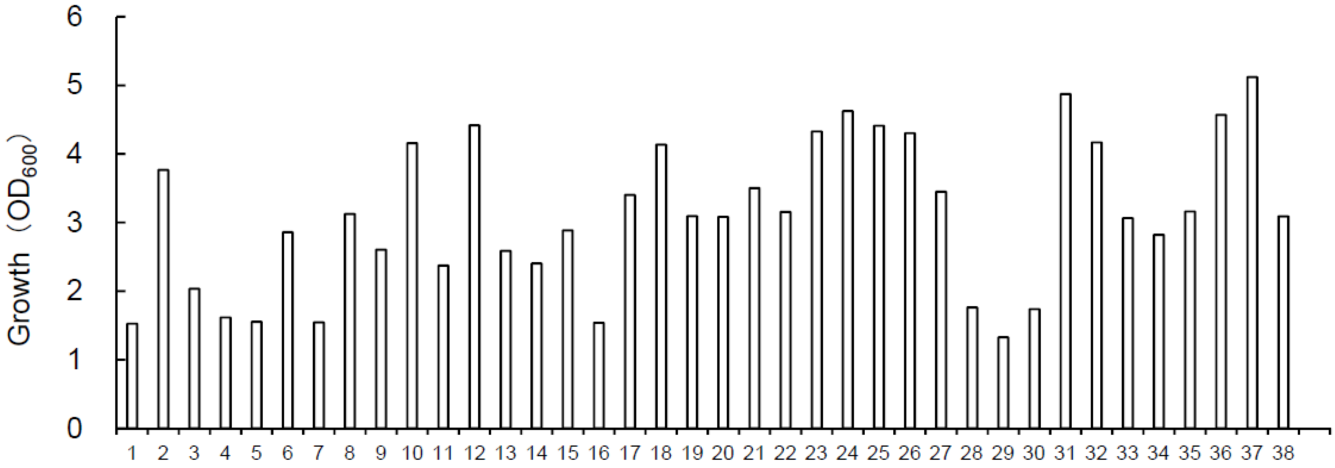

Figure S1. Growth of bacterial strains isolated from the blue cheese in MRS broth. Bacterial strains isolated from the blue cheese was inoculated in 500 μL of MRS broth and incubated under anaerobic conditions for 48 h at 37 °C. At the end of the cultivation, the OD_{600} values were measured.

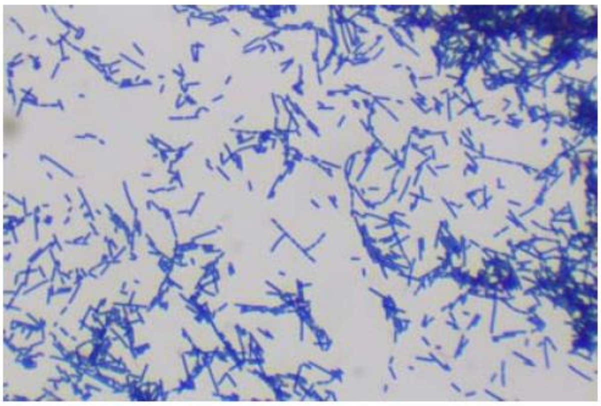

Figure S2. Gram-stained photograph of *L. brevis* FB215.

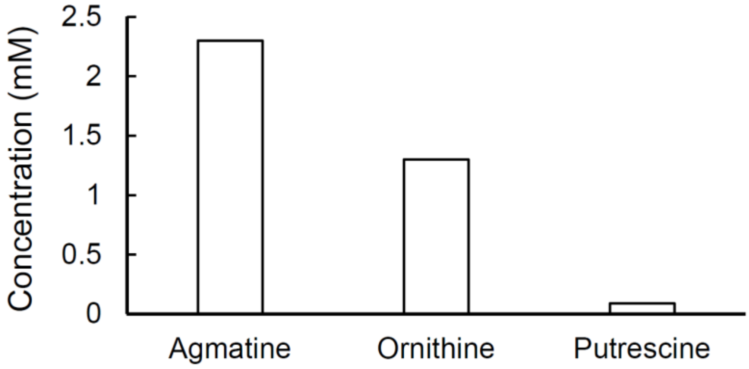

Figure S3. Concentration of polyamine precursors (ornithine and agmatine) and putrescine in Sakekasu extract. Ornithine, agmatine, and putrescine concentrations in the Sakekasu extract were determined by HPLCs.

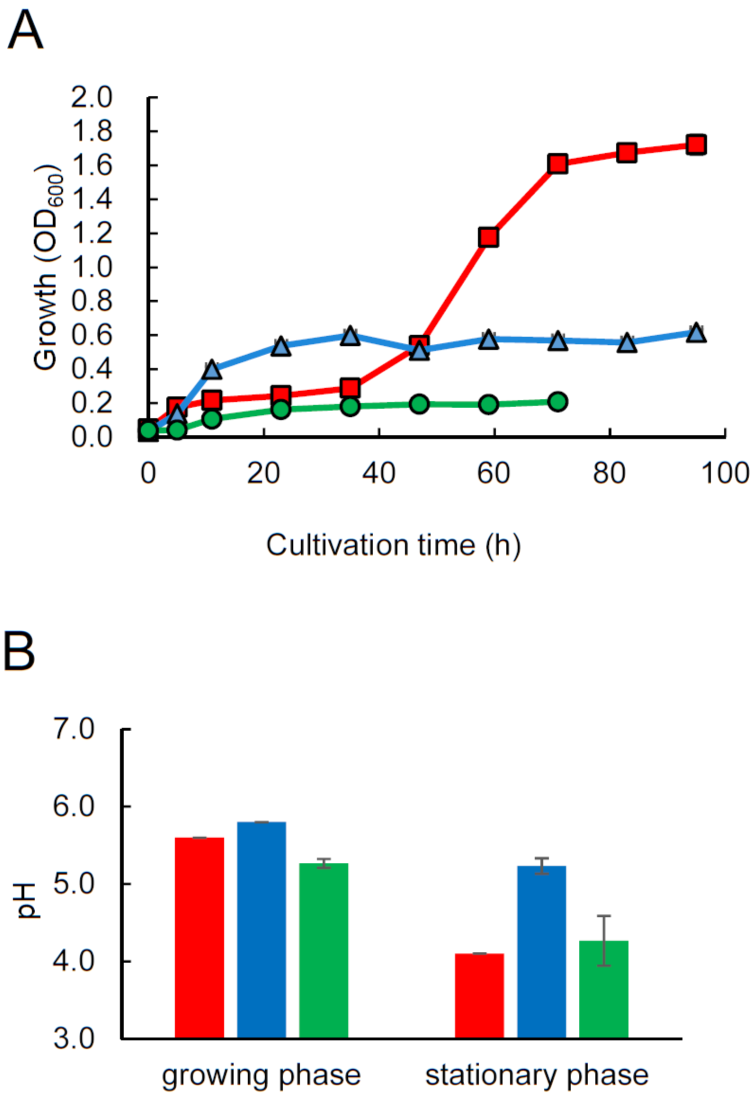

Figure S4. Growth of high polyamine-producing bacteria isolated from fermented foods grown in Sakekasu extract. *L. brevis* FB215, *S. epidermidis* FB146 [1], and *L. curvatus* KP3-4 [2] were inoculated in Sakekasu extract and incubated at 37 °C. (**A**)The OD_{600} values of cultures at different growth phases were determined. (**B**) The pH of the different growth phases was measured. The pH of culture supernatant of *L. brevis* FB215, *S. epidermidis* FB146, and *L. curvatus* KP3-4 are represented by red, blue, and green bars, respectively (n = 3).

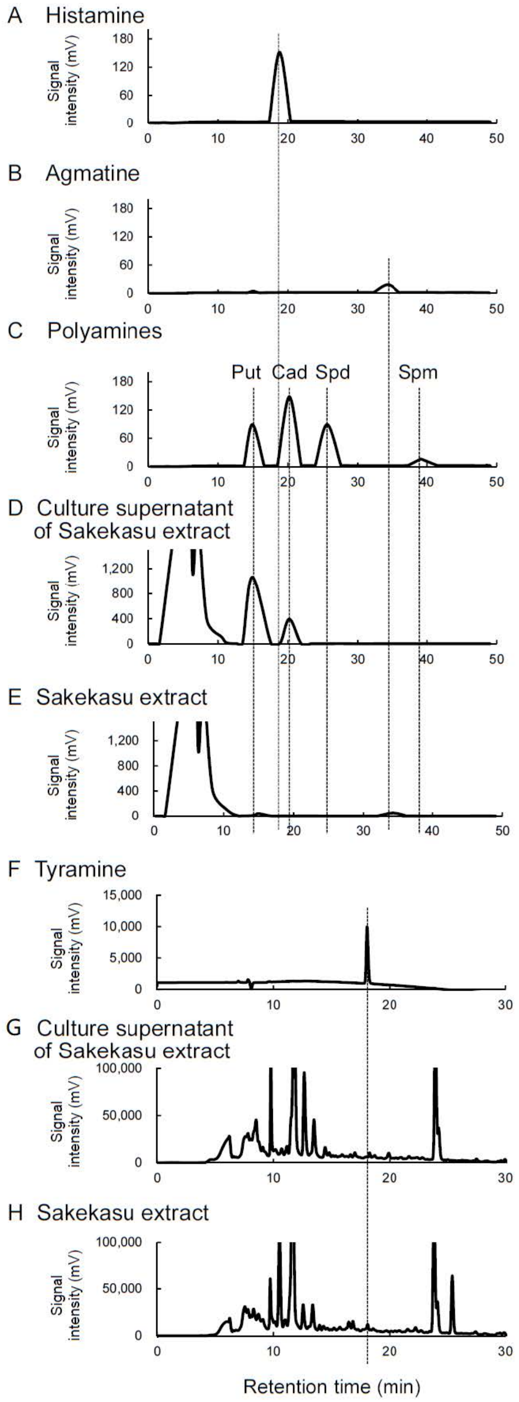

Figure S5. Analysis of metabolites in the supernatant of *L. brevis* FB215 cultured in Sakekasu extract. (**A**–**C**) show chromatograms of 100 μM histamine, 100 μM agmatine and 100 μM polyamines, respectively. In (**C**), Put, Cad, Spd and Spm represent putrescine, cadaverine, spermidine and spermine, respectively. (**D**) and (**E**) are chromatograms of the culture supernatant of *L. brevis* FB215 cultured in Sakekasu extract for 96 hours and Sakekasu extract before inoculation, respectively. In (**A**–**E**), the HPLC system equipped with the #2619PH column was used. (**F**–**H**) show chromatograms of 100 μM tyramine, culture supernatant of *L. brevis* FB215 cultured in Sakekasu extract, and Sakekasu extract before inoculation, respectively. In (**F**–**H**), the HPLC equipped with the Discovery HS F5 column was used.

Reference

1. Shirasawa, H.; Nishiyama, C.; Hirano, R.; Koyanagi, T.; Okuda, S.; Takagi, H.; Kurihara, S. Isolation of the high polyamine-producing bacterium Staphylococcus epidermidis FB146 from fermented foods and identification of polyamine-related genes. *Biosci. Microbiota, Food Heal.* **2023**, *42*, 24–33, https://doi.org/10.12938/bmfh.2022-011.
2. Hirano, R.; Kume, A.; Nishiyama, C.; Honda, R.; Shirasawa, H.; Ling, Y.; Sugiyama, Y.; Nara, M.; Shimokawa, H.; Kawada, H.; et al. Putrescine Production by *Latilactobacillus curvatus* KP 3-4 Isolated from Fermented Foods. *Microorganisms* **2022**, *10*, 697, https://doi.org/10.3390/microorganisms10040697.
